# Osteocyte-Specific Deletion of *Fgfr1* Suppresses FGF23

**DOI:** 10.1371/journal.pone.0104154

**Published:** 2014-08-04

**Authors:** Zhousheng Xiao, Jinsong Huang, Li Cao, Yingjuan Liang, Xiaobin Han, Leigh Darryl Quarles

**Affiliations:** University of Tennessee Health Science Center, Memphis, Tennessee, United States of America; Nihon University School of Medicine, Japan

## Abstract

Increases in fibroblastic growth factor 23 (FGF23 or Fgf23) production by osteocytes result in hypophosphatemia and rickets in the *Hyp* mouse homologue of X-linked hypophosphatemia (XLH). Fibroblastic growth factor (FGF) signaling has been implicated in the pathogenesis of *Hyp*. Here, we conditionally deleted FGF receptor 1 (*FGFR1* or *Fgfr1*) in osteocytes of *Hyp* mice to investigate the role of autocrine/paracrine FGFR signaling in regulating FGF23 production by osteocytes. Crossing dentin matrix protein 1 (*Dmp1*)-Cre;*Fgfr1* ^null/+^ mice with female *Hyp*;*Fgfr1* ^flox/flox^ mice created *Hyp* and *Fgfr1* (*Fgfr1^Dmp1-cKO^*)-null mice (*Hyp*;*Fgfr1* ^Dmp1-*cKO*^) with a 70% decrease in bone *Fgfr1* transcripts. *Fgfr1^Dmp1-cKO^*-null mice exhibited a 50% reduction in *FGF23* expression in bone and 3-fold reduction in serum FGF23 concentrations, as well as reductions in *sclerostin* (*Sost)*, phosphate regulating endopeptidase on X chromosome *(PHEX or Phex)*, matrix extracellular phosphoglycoprotein (*Mepe*), and *Dmp1* transcripts, but had no demonstrable alterations in phosphate or vitamin D homeostasis or skeletal morphology. *Hyp* mice had hypophosphatemia, reductions in 1,25(OH)_2_D levels, rickets/osteomalacia and elevated FGF2 expression in bone. Compared to *Hyp* mice, compound *Hyp*;*Fgfr1* ^Dmp1*-*cKO^-null mice had significant improvement in rickets and osteomalacia in association with a decrease in *s*erum FGF23 (3607 to 1099 pg/ml), an increase in serum phosphate (6.0 mg/dl to 9.3 mg/dl) and 1,25(OH)_2_D (121±23 to 192±34 pg/ml) levels, but only a 30% reduction in bone *FGF23* mRNA expression. FGF23 promoter activity in osteoblasts was stimulated by FGFR1 activation and inhibited by overexpression of a dominant negative FGFR1(TK−), PLCγ and MAPK inhibitors. FGF2 also stimulated the translation of an FGF23 cDNA transfected into osteoblasts via a FGFR1 and PI3K/Akt-dependent mechanism. Thus, activation of autocrine/paracrine FGF pathways is involved in the pathogenesis of *Hyp* through FGFR1-dependent regulation of FGF23 by both transcriptional and post-transcriptional mechanisms. This may serve to link local bone metabolism with systemic phosphate and vitamin D homeostasis.

## Introduction

The FGF family consists of canonical FGFs, intracellular FGFs, and hormone-like FGF gene products (i.e., FGF19, FGF21 and FGF23). The earliest evolved family members are likely intracellular FGFs, exemplified by high molecular weight FGF2 (HMW-FGF2), which interact with intranuclear FGFR1 to directly activate gene transcription (*i.e.*, integrative nuclear FGFR1 signaling) [1]. Canonical or FGFs evolved later to serve autocrine/paracrine functions. These secreted FGFs have high heparin affinity, limited diffusion capacity and act locally on cell surface FGF receptors [2]. More recently, circulating FGFs emerged with the capability to diffuse from tissues and specifically target FGF receptor/Klotho complexes at distal sites due to their unique C-terminal domain [3]. For example, FGF23 is a ∼32 kDa hormone with an N-terminal FGF-homology domain and a novel 71 amino acid C-terminus [4] that allows it to be released into the circulation and to interact with α-Klotho, a type I membrane, β-glycosidase to form a trimeric complex with FGFRs in specific tissues [4]–[9].

FGF23 is produced and secreted by osteoblasts/osteocytes in bone. FGF23 activates FGFR/α-Klotho complexes in the kidney to decrease Npt2a co-transporters leading to inhibition of renal tubular phosphate reabsorption and to reduce circulating 1,25(OH)_2_D levels by inhibiting enzymes regulating vitamin D metabolism (i.e., inhibiting *Cyp27b1* and stimulating *Cyp24*) [6], [7], [9]–[13]. The biological functions of FGF23 are essential for maintenance normal mineral metabolism. Ablation of *FGF23* in mice is lethal in the early postnatal period due to hyperphosphatemia and excessive 1,25(OH)_2_D production [14], [15]. On the other hand, **e**xcess FGF23 causes hypophosphatemia, aberrant vitamin D metabolism and rickets/osteomalacia. Increases in FGF23 underlie acquired and hereditary forms of hypophosphatemic rickets and are involved in the pathogenesis of mineral metabolism abnormalities in chronic kidney diseases [16]. Consequently, understanding the factors that regulate FGF23 is of high clinical importance.

FGF23 gene transcription in bone is complex and poorly understood. FGF23 is regulated by systemic factors, including 1,25-(OH)_2_D, PTH, and calcium [17] and local bone derived factors [14], [17]–[29]. Systemic regulators have variable and often opposite effects on FGF23 expression that are possibly explained by their differential effects on bone mineralization and/or the presence of hypocalcaemia. Local bone-derived factors that regulate bone mineralization are important regulators of FGF23 expression, although the mechanisms are poorly understood. For example, high circulating FGF23 and increased *FGF23* gene transcription occur in XLH rickets and its *Hyp* mouse homologue. XLH and Hyp are caused by inactivating mutations of *PHEX*, one of several genes regulating bone mineralization and FGF23 production. Disruption of the extracellular matrix (ECM) mineralization by mutations of *DMP1*, *PHEX*, and *ENPP1* leads to increased FGF23 gene expression in osteoblasts/osteocytes [29]–[33]. How these mutations lead to elevated FGF23 remains unclear.

Recent studies implicate a role of FGFR1 activation in regulating FGF23 gene transcription [29], [34]–[36]. In this regard, FGF23 is increased in osteoglophonic dysplasia, which is caused by activating mutations in *FGFR1* [35], [36]. In addition, ligands for FGFR1, including FGF1, FGF2, and FGF7 are significantly increased in the *Hyp* and *Dmp1* knockout mice [37]–[39]. Pharmacological inhibition of FGFR1 also blocks FGF23 transcription in bone both *in vitro* and *ex vivo* [29], [40], [41]. Moreover, recent studies have reported that administration of monoclonal FGFR1 activating antibodies stimulates FGF23 production and induces hypophosphatemia [42]. These findings suggest that autocrine/paracrine FGF/FGFR1 signaling pathways may be involved in regulation of hormonal FGF23. The gain-of-function studies implicating FGFR1 in the regulation of FGF23, however, are confounded by the generalized effects to activate FGF receptors in multiple tissues. Interpretation of the inhibitor studies are confounded by actions to block FGF23 end-organ effects and other potential systemic actions that could lead to feed-back regulation of FGF23. These limitations preclude establishing a direct cause and effect relationship between FGFR1 function and FGF23 gene transcription in osteoblasts/osteocytes.

In the current study, to test the central role of FGFR1 in mediating the increased expression of FGF23 in bone, we use a mouse genetic approach to conditionally deleted *Fgfr1* from osteocytes of *Hyp* mice. We find an important role of FGFR1 signaling in osteocytes in mediating the increase of FGF23 caused by *Phex* mutations in *Hyp* mice, thereby linking alterations autocrine/paracrine functions of FGF/FGFR1 pathways in the bone microenvironment with the secretion of circulating FGF23 that activates FGFRs in distal tissues to coordinate bone mineralization with renal regulation of phosphate and vitamin D metabolism.

## Materials and Methods

### Animals breeding and genotyping

All animal research was conducted according to guidelines provided by the National Institutes of Health and the Institute of Laboratory Animal Resources, National Research Council. The University of Tennessee Health Science Center's Animal Care and Use Committee approved all animal studies (Protocol number: 12-162.0). *CMV-*Cre and *Hyp* mice were originally purchased from the Jackson Laboratory (Bar Harbor, ME, USA) and maintained in C57BL/6J background. The floxed *Fgfr1* mice (*Fgfr1^flox/+^*) were obtained from Dr. Chuxia Deng at National Institute of Diabetes and Digestive and Kidney Diseases and maintained in C57BL/6J background for at least six generations. 9.6-kb *Dmp1-*Cre mice were used to delete the floxed *Fgfr1* in bone as described previously [43] and maintained in C57BL/6J background for at least five generations. The 9.6-kb *Dmp1*-Cre has been widely utilized to delete a gene specifically in osteocytes [43], [44], where it has been shown to be active in 77% of osteocytes and only ∼1% of surface osteoblasts in long bone [45], [46]. All mice were maintained on a standard diet (7912, Harlan Teklad, Madison, WI, USA). First, we crossed *Fgfr1^flox/+^* to *CMV*-Cre to obtain a germline-specific deletion of *Fgfr1* (*Fgfr1* ^null/*+*^). The *Fgfr1* ^null/*+*^ mice were crossed to *Dmp1-*Cre mice to obtain double heterozygous *Dmp1-*Cre;*Fgfr1* ^null/*+*^ mice. Second, heterozygous female *Hyp* (XX^Hyp^) mice were crossed to *Fgfr1* ^flox/flox^ males to obtain *Hyp*;*Fgfr1* ^flox/+^ double heterozygous females. Then *Hyp*;*Fgfr1* ^flox/+^ females were crossed to *Fgfr1* ^flox/flox^ males to generate *Hyp*;*Fgfr1* ^flox/flox^ females. Finally, *Hyp*;*Fgfr1* ^flox/flox^ females were crossed to *Dmp1-*Cre;*Fgfr1* ^null/*+*^ males to obtain the osteocyte-specific deletion of *Fgfr1* in *Hyp* mice. These mice are on a mixed genetic background. For the entire study, samples were collected from 6-week-old *Fgfr1^flox/+^* (wild-type equivalent) control, conditional *Dmp1-*Cre;*Fgfr1* ^flox/+^ heterozygous, conditional *Dmp1-*Cre;*Fgfr1* ^null/flox^ (*Fgfr1* ^Dmp1-cKO^)-null, *Hyp* (X^Hyp^Y), and compound *Hyp*;*Fgfr1* ^Dmp1-cKO^-null male littermates as well as *Fgfr1* ^flox/null^ (equivalent to *Fgfr1* ^+/null^) and *Hyp*;*Fgfr1* ^flox/null^ (equivalent to *Hyp*;*Fgfr1* ^+/null^) male mice. Tail clips were collected to genotype the mice. REDExtract-N-Amp Tissue PCR Kit (Sigma-Aldrich, St. Louis, MO, USA) was used for DNA extraction and PCR amplification. Mice were genotyped for *Phex* mutations and *Dmp1-*Cre using previously described primers [43], [47], for the *Fgfr1^flox^* allele using forward primer 5′-CTG GTA TCC TGT GCC TAT C-3′ and reverse primer 5′-CCA ATC TGA TCC CAA GAC CAC-3′ (325 bp product for the *Fgfr1* ^+^ wild-type allele, 400 bp product for the *Fgfr1* ^flox^ floxed allele), and for the *Fgfr1* ^null^ allele using forward primer 5′-GTA TTG CTG GCC CAC TGT TC-3′ and reverse primer 5′-CCA ATC TGA TCC CAA GAC CAC-3′ (250 bp product for the *Fgfr1* ^null^ null allele).

### Serum Biochemistry

Serum samples were collected by intracardiac exsanguinations and urine samples were collected overnight in metabolic cages. Calcium was measured using a Calcium CPC Liquicolor Kit (Stanbio Laboratories, Boerne, TX, USA) and phosphorus was measured using the phosphomolybdylate-ascorbic acid method, as previously described [47]. Serum parathyroid hormone (PTH) levels were measured using the Mouse Intact PTH ELISA kit (Immutopics, Carlsbad, CA, USA). Serum full-length FGF23 levels were measured using the FGF23 ELISA kit (Kainos Laboratories, Tokyo, Japan), and serum C-terminal Fgf23 levels were measured using the FGF23 (C-Term) ELISA kit (Immutopics, Carlsbad, CA, USA).

### Bone and kidney RNA isolation and real-time reverse transcriptase (RT)-PCR

For quantitative real-time RT-PCR, 1.0 µg total RNA isolated from tibias without bone marrow and kidney of 6-week-old four genotypes mice was reverse transcribed as previously described [48]. PCR reactions contained 20 ηg template (cDNA or RNA), 375 ηM each forward and reverse primers, and 1× SsoFast EvaGreen supermix (Bio-Rad, Hercules, CA, USA) in a total of 10 µl reaction volume. The threshold cycle (Ct) of tested gene product from the indicated genotype was normalized to the Ct for cyclophilin A. Expression of total *Fgfr1* transcripts was performed using the following *Fgfr1-*allele-specific primers in exons 9 and 10: forward primer of normal *Fgfr1* ^+^ transcripts (*Fgfr1* ^+^ plus *Fgfr1* ^flox^): 5′ – ACC AAG AAG AGC GAC TTC CA -3′ and reverse primer: 5′ – AAC CAG GAG AAC CCC AGA GT -3′. The normal *Fgfr1* ^+^ vs. cyclophilin A was normalized to the mean ratio of five control mice, which was set to 1. The percentage of *Fgfr1* null (*Fgfr1* ^null^) and/or conditional deleted (*Fgfr1* ^Δflox^) transcripts was calculated from the relative levels of the normal *Fgfr1* ^+^ transcripts in different *Fgfr1*-deficient mice [49]. All primer information of other genes used in real-time RT-PCR can be found in our previous report [44].

### High resolution 3D microtomography

The femurs were collected, fixed and dehydrated in 70% ethanol. High-resolution micro-Computed Tomography (μCT40, Scanco Medical, Basserdorf, Switzerland) was used to scan and evaluate the metaphyseal trabecular bone microarchitecture and the midshaft cortical bone parameters. The entire femurs were scanned in a 12.3 mm diameter sample holder at 6 µm resolution: energy level of 55 KeV and intensity of 145 µA. Evaluation of the bone growth was obtained by measuring the length of the scanned femur. The trabecular bone volume (BV/TV%) was measured within the secondary spongiosa on a set of 50 sections (0.6 mm) underneath the growth plate at a threshold of 200 as previously described [50]. The cortical bone thickness (CtTh, mm) was analyzed from 100 sections chosen at the midshaft of each femur at a threshold of 350.

### Bone histology and histomorphometry

Evaluation of the bone growth was obtained by measuring the length of the femur of 6-week-old mice with a slide caliper. Femurs were fixed and dehydrated in 70% ethanol, and embedded in methylmetacrylate at low temperature. Nonserial longitudinal frontal slices (5 µm) were cut from the embedded bones with a microtome (Polycut-S; Reichert-Jung, Wetzlar, Germany) and were either left unstained or used for modified Goldner staining. Alizarin complexone dehydrate (Sigma, St. Louise, MO) double labeling of bone and histomorphometric analyses of periosteal mineral apposition rate (MAR) in femurs were performed using the osteomeasure analysis system (OsteoMetrics, Decatur, GA, USA).

### *In vitro* promoter studies

We used the MC3T3-E1 osteoblastic cell line from Dr. Hiroko Sudo [51] that we previously characterized [52]. MC3T3-E1 osteoblasts were maintained in α-MEM containing 10% fetal bovine serum (FBS) and 1% penicillin and streptomycin (P/S). To perform plasmid transfection in MC3T3E1 cells, 5×10^4^ cells were seeded in 6-well plates in α-MEM media (Life technologies, Grand Island, NY) with 10% FBS at 37°C in 5% CO_2_ and humidified incubator. Cells were plated for 18 hours before transfection and fed with fresh medium 4 hours before transfection. Mouse 0.6 kb FGF23 promoter luciferase reporter construct (0.25 µg) along with pcDNA3.1-*FGFR1* cDNA expression plasmids (0.5 µg) or pcDNA3.1-*FGFR1(TK−)* cDNA expression plasmids (0.5 µg) or HMW-*FGF2* (0.25 µg) (a gift from Dr. Michal K. Stachowiak at Neuroscience State University of New York at Buffalo) [53] and pRL *Renilla reniformis*luciferase control plasmids (0.1 µg) were co-transfected into MC3T3-E1 cells using cationic liposomes (LipofectAMINE2000, Life technologies, Grand Island, NY).for 16–18 hours, and then cells were washed twice with phosphate-buffered saline and incubated in fresh medium containing 10% FBS for 38 hours. Various doses of FGF2 (0∼100 ng/ml) or FGF2 (50 ng/ml) in the presence and absence of Wortmannin (PI3K inhibitor, 1.0 µM), various doses of U73122 (PLC inhibitor, 1∼10 µM), and various doses of U0126 (MEK inhibitor, 5∼20 µM) were added to the cell culture media for 24 hours before cells were harvested. Cells were lysed in 50 µl of reporter lysis buffer (Promega, Madison, WI). A luciferase assay (20 µl of cell lysed) was performed using a dual luciferase assay kit (Promega, Madison, WI), and activity was measured with an Optocomp 1 luminometer (MGM Instruments, Inc., Hamden, CT).

### Western blot analysis

Bone marrow-free femurs from four groups of mice were homogenized into a fine powder in liquid nitrogen using a porcelain mortar and pestle. The powder was transferred into T-PER Tissue Protein Extraction Reagent with 1× Halt protease inhibitor (Thermo Scientific, Rockford, IL) and 1 mM phenylmethylsulfonyl fluoride (PMSF). After three 30-second sonications, samples were centrifuged at 13,000× *g* for 10 minutes and supernatants were stored at −80°C until use.

To examine if Fgfr1 signaling has a role in translational control of FGF23 protein expression *in vitro*, a number of 1.2×10^6^ of MC3T3-E1 cells were transfected with pcDNA3.1-*FGFR1* cDNA expression plasmids (3.0 µg) and human pcDNA3.1-FGF23-V5-His cDNA expression plasmids (3.0 µg) conducted by electroporation using Cell Line Nucleofector Kit R according to the manufacturer's protocol (Amaxa Inc, Gaithersburg, MD). 2.0×10^5^ cells were seeded in 6-well plates in α-MEM media (Life technologies, Grand Island, NY) with 10% FBS at 37°C in 5% CO_2_ and humidified incubator for 8 hours. For time-course experiments, the cells were changed into fresh growth medium containing 5% FBS and FGF2 (50 ng/ml, Sigma-Aldrich, St. Louis, MO) and heparin (10 µg/ml, Sigma-Aldrich, St. Louis, MO) were added to the cell culture media for 8, 24. And 48 hours before cells were harvested. For signaling mechanism study, the cells were changed into fresh growth medium containing 5% FBS and FGF2 (50 ng/ml) and heparin (10 µg/ml) in the presence and absence of Wortmannin (PI3K inhibitor, 1.0 µM) or Cycloheximide (0.5 µg/ml, Sigma-Aldrich, St. Louis, MO) were added to the cell culture media for 24 hours before cells were harvested. The cells were lysed with 150 µl of T-PER with 1× Halt protease inhibitor and 1 mM per well, After three 30-second sonications, total cell lysates were centrifuged at 13,000× *g* for 10 minutes and supernatants were stored at −80°C until use. Protein concentrations of the supernatant were determined with a Bio-Rad protein assay kit (Bio-Rad, Hercules, CA). Equal quantities of protein were subjected to NuPAGE 4–12% Bis-Tris Gel (Invitrogen, Carlsbad, CA) and were analyzed with standard Western blot protocols (HRP-conjugated secondary antibodies from Santa Cruz Biotechnology and ECL from GE Healthcare Bio-Sciences (Pittsburgh, PA). Antibody against Fgf23 (MAB2629) was obtained from R&D Systems, Inc. (Minneapolis, MN). Antibody against Fgf2 (610073) was purchased from BD Biosciences (San Jose, CA). Anti-V5-HRP antibody (R961-25) was purchased from Life technologies (Carlsbad, CA). Anti-β-actin (sc-47778) antibodies were from Santa Cruz Biotechnology (Paso Robles, CA). The intensity of bands was quantified using Image J software (http://rsb.info.nih.gov/ij/).

### Statistics

We evaluated differences between two groups by unpaired t-test and multiple groups by one-way analysis of variance. All values are expressed as means ± SD. All computations were performed using GraphPad Prism5 (GraphPad Software Inc. La Jolla, CA, USA).

## Results

### Osteocyte specific deletion of *Fgfr1* in mice

We selectively deleted *Fgfr1* in osteocytes by crossing *Dmp1*-Cre;*Fgfr1* ^+/null^ mice with homozygous *Hyp*;*Fgfr1* ^flox/flox^ mice. Conditional *Fgfr1* ^Dmp1-cKO^ (or *Dmp1*-Cre;*Fgfr1* ^flox/null^)-null mice with intact *Phex* but reduced *Fgfr1* in osteocytes; *Hyp* (*X^Hyp^Y*) mice with intact *Fgfr1* but mutant *Phex*; compound *Hyp* (*X^Hyp^Y*);*Dmp1*-Cre;*Fgfr1* ^flox/null^ mice with reduced *Fgfr1* in osteocytes and mutant *Phex*, hereafter referred to as *Hyp*;*Fgfr1* ^Dmp1-cKO^-null mice; and *Fgfr1^flox/+^* control mice with both intact *Fgfr1* and *Phex*, were all born with the expected Mendelian frequency. There was no difference in survival between genotypes produced by this breeding strategy. All mice survived to adulthood.

**Figure 1:**
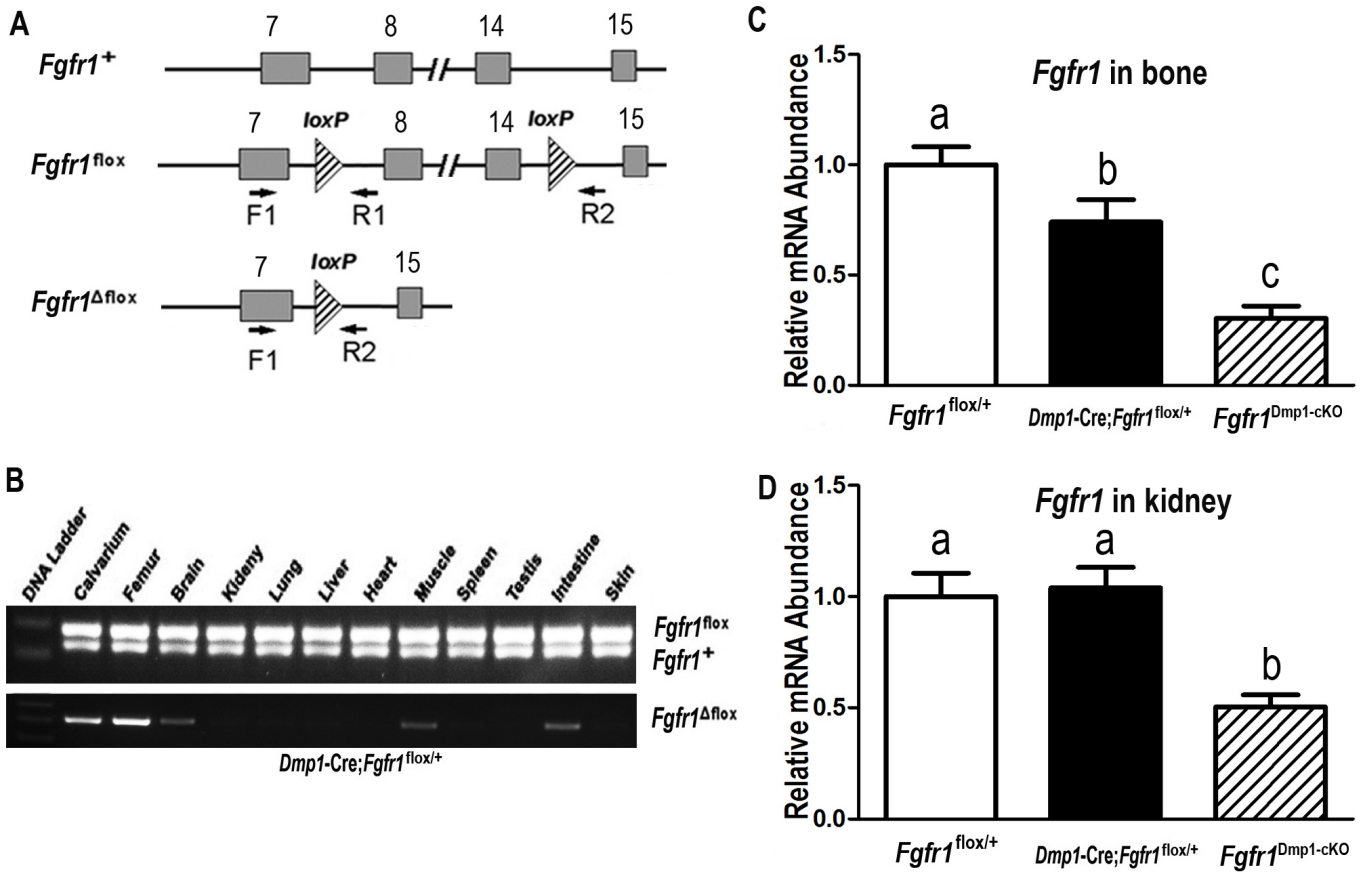
*Dmp1*-Cre-mediated conditional deletion of *Fgfr1* from the floxed *Fgfr1* allele (*Fgfr1* ^flox^) in different tissues. (A) Schematic illustration of wild-type (*Fgfr1* ^+^), floxed *Fgfr1* allele before (*Fgfr1* ^flox^) and after deletion (*Fgfr1* ^Δflox^) of the lox P cassette containing Exon 8–14 via Cre-mediated recombination. “//” stands for all the Introns and Exons omitted between Exon 8 and Exon 14. (B) Genotype PCR analysis of different tissues that were harvested from heterozygous *Dmp1*-Cre;*Fgfr1* ^flox/+^ mice at 6 weeks of age. Both *Fgfr1* wild-type and floxed alleles existed in all tested tissues of heterozygous *Dmp1*-Cre;*Fgfr1* ^flox/+^ mice. However, *Dmp1*-Cre-mediated recombination of excised floxed *Fgfr1* (*Fgfr1* ^Δflox)^ allele occurred in bone tissues such as calvarias and femur, but also had a leakage in the brain, muscle, and intestine. (C and D) Real-time RT-PCR analysis of total *Fgfr1* transcripts in bone and kidney. Expression of total *Fgfr1* transcripts was performed using *Fgfr1-*allele-specific primers as described in Materials and Methods. The normal *Fgfr1* ^+^ vs cyclophilin A is normalized to the mean ratio of 5 control *Fgfr1* ^flox/+^ mice, which has been set to 1. Data are expressed as a relative abundance of wild-type (*Fgfr1* ^+^ and *Fgfr1* ^flox^) mRNA messages in control *Fgfr1* ^flox/+^ mice, heterozygous *Dmp1*-Cre;*Fgfr1* ^flox/+^, and homozygous *Dmp1*-Cre;*Fgfr1* ^flox/null^ (*Fgfr1* ^Dmp1-cKO^)-null mice. Values sharing the same superscript in different groups are not significantly different at *P*<0.05.

To confirm *Dmp1*-Cre-mediated conditional deletion of *Fgfr1* in bone, we performed PCR analysis of genomic DNA using a combination of primers that specifically detect floxed *Fgfr1* alleles (*Fgfr1* ^flox^) and the excised floxed *Fgfr1* alleles (*Fgfr1* ^Δflox^), as well as wild type alleles (*Fgfr1* ^+^) in *Dmp1*-Cre;*Fgfr1* ^flox/+^ heterozygous mice (Figure 1A). We found that *Dmp1*-Cre-mediated excision of the *Fgfr1* ^Δflox^ alleles occurred in bone tissues, including calvaria and femur. Low levels of recombinase activity were detected in brain, muscle, and intestine, indicating that *Dmp1*-Cre promoter is not restricted to bone (Figure 1B). We found no evidence for the *Fgfr1* ^Δflox^ alleles in a wide range of other tissues tested, including the kidney. The floxed *Fgfr1* ^flox^ alleles were detected in all tissues tested (Figure 1B). Real-time RT-PCR analysis revealed that *Fgfr1* mRNA levels in bone tissues were reduced by about 30% from conditional *Dmp1*-Cre;*Fgfr1* ^flox/+^ mice and about 70% from conditional *Fgfr1* ^Dmp1-cKO^-null mice compared with control mice (Figure 1C), respectively. Consistent with the absence of *Dmp1*-Cre expression in kidney, there were no reduction in *Fgfr1* transcripts in the kidney of *Dmp1*-Cre;*Fgfr1* ^flox/+^, but *Fgfr1* transcripts were reduced by 50% in *Fgfr1* ^Dmp1-cKO^-null mice, consistent with the presence of a null *Fgfr1* allele (Figure 1D).

### Effects of osteocyte specific deletion of *Fgfr1* on mouse bone phenotype

**Figure 2:**
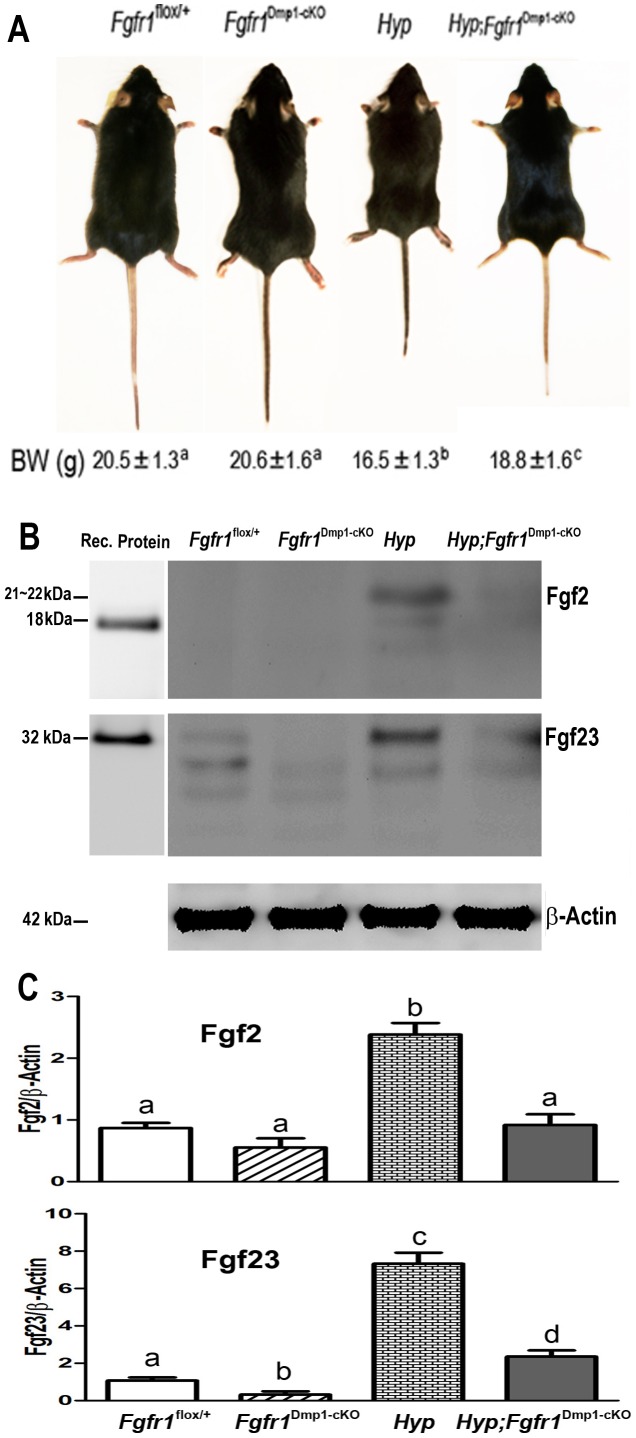
Effects of conditional deletion of *Fgfr1* in *Hyp* osteocytes on gross appearance and bone-related gene expressions in 6-week-old mice. (A) Gross appearance, tail length, and body weight. Compared with control mice, *Fgfr1* ^Dmp1-cKO^-null mice had normal gross appearance and body weight. However, *Hyp* mice showed considerably shorter tail length and lower body weight, compound *Hyp*;*Fgfr1* ^Dmp1-cKO^-null mice displayed intermediate tail length and body weight between control and *Hyp* mice. Data are mean ± S.D. from 5–6 individual mice. (B and C) Western blot analysis of total Fgf2 and Fgf23 protein levels in bone. A representative Fgf2, Fgf23, and β-Actin gel were shown in upper, middle, and lower panels of B, respectively. The intensity of bands was quantified using Image J software (http://rsb.info.nih.gov/ij/), and the data shown in C are mean ± S.D. from three independent experiments. Values sharing the same superscript in different groups are not significantly different at *P*<0.05.

**Figure 3:**
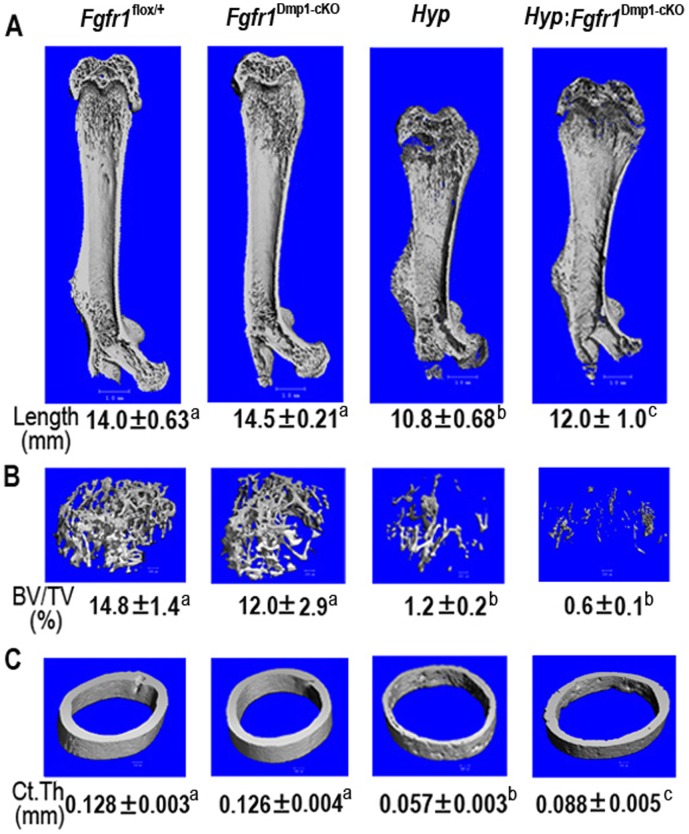
Effects of conditional deletion of *Fgfr1* in *Hyp* osteocytes on bone structure in 6-week-old mice. Representative μCT 3D images of (A) whole femur segital section, (B) the distal femoral metaphyses, and (C) femoral midshaft diaphyses in four genotypes of mice. Compared with control mice, *Fgfr1* ^Dmp1-cKO^-null mice had normal bone structure. However, *Hyp* mice showed considerably shorter femur length, less trabecular bone volume, and thinner cortical thickness, compound *Hyp*;*Fgfr1* ^Dmp1-cKO^-null mice displayed intermediate femur length and cortical thickness between control and *Hyp* mice, but no recovery was found in trabecular bone volume. Data are mean ± S.D. from 5–6 individual mice. Values sharing the same superscript in A, B, and C are not significantly different at *P*<0.05.

**Figure 4:**
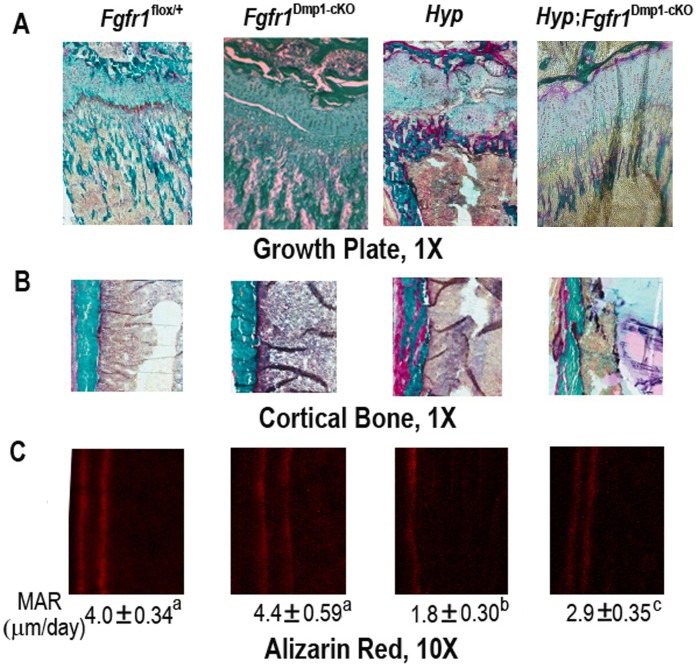
Effects of conditional deletion of *Fgfr1* in *Hyp* osteocytes on bone histology in 6-week-old mice. Representative images of (A) the distal femoral growth plate, (B) the femoral cortical bone by Goldner staining, and (C) femoral segital section by Alizarin Red double labeling in four genotypes of mice. Compared with control mice, *Fgfr1* ^Dmp1-cKO^-null mice had normal growth plate, no osteomalacia in cortical bone, and normal mineral apposition rate. However, *Hyp* mice showed a unorganized growth plate, an apparent increase of un-mineralized osteoid in both trabecular and cortical bone areas, and significant reduction in mineral apposition rate, compound *Hyp*;*Fgfr1* ^Dmp1-cKO^-null mice exhibited a recovery of organized growth plate, only fewer un-mineralized osteoid in cortical bone, and increased mineral apposition rate between control and *Hyp* mice, suggesting a partial rescue of the hypophosphatemic rickets phenotype in the compound *Hyp*;*Fgfr1* ^Dmp1-cKO^-null mice. Data are mean ± S.D. from 5–6 individual mice. Values sharing the same superscript in C are not significantly different at *P*<0.05.

*Dmp1*-Cre;*Fgfr1* ^flox/+^ mice were indistinguishable from *Fgfr1^flox/+^* littermates and wild-type mice. We focused our studies on *Fgfr1* ^Dmp1-cKO^-null and compound *Hyp*;*Fgfr1* ^Dmp1-cKO^-null mice. The overall appearance, body weight and femur length of *Fgfr1* ^Dmp1-cKO^-null mice were indistinguishable from *Fgfr1^flox/+^* littermates, which we used as controls (Figure 2A and 3A). In spite of the known role of Fgfr1 in bone development [54], conditional deletion of *Fgfr1* in osteocytes, which represent the terminal stage of osteoblasts differentiation, had minimal effects on the skeletal phenotype. Micro-CT 3D image analyses of the femur revealed that deletion of *Fgfr1* in osteocytes resulted in no measurable changes in cortical or trabecular bone structural parameters. Indeed, *Fgfr1* ^Dmp1-cKO^-null mice had normal femur length, trabecular bone volume, and cortical thickness (Figure 3). The histological appearance of bone was indistinguishable in *Fgfr1* ^Dmp1-cKO^-null mice (i.e., normal growth plate, trabecular bone and osteoid volume and mineral apposition rate) compared with age-matched control mice (Figure 4).

Assessment of mRNA expression in bone, however, revealed that the conditional deletion of *Fgfr1* in osteocytes did result in significant but selective reductions in osteocyte-specific gene expression, including *Fgf23*, *Sost*, *Phex*, *Dmp1*, and *Mepe*, but not *Osteopontin, Osteocalcin, Fgf1, Fgf2, Fgfr2, Fgfr3, Fgfr4, and Mmp13* (Table 1). Consistent with the 50% decrease of *Fgf23* transcripts, western blot analysis showed a significant reduction of FGF23 protein in bone from *Fgfr1* ^Dmp1-cKO^-null mice (Figure 2B and 2C). Conditional deletion of *Fgfr1* in osteocytes also resulted in a 2-fold reduction in serum FGF23 protein levels in the *Fgfr1* ^Dmp1-cKO^-null mice (Table 2).

**Table 1:** Gene-expression profiles in bone in 6-week-old mice.

Gene	*Fgfr1* ^flox/+^	*Fgfr1* ^Dmp1-cKO^	*Hyp*	*Hyp;Fgfr1* ^Dmp1-cKO^	*p*-value
**Osteoblast lineage**					
*Fgfr1*	1.00±0.12^a^	0.30±0.15^b^	1.64±0.12^c^	0.31±0.14^b^	<0.0001
*Fgfr2*	1.00±0.22^a^	0.86±0.29^a^	1.87±0.77^b^	1.02±0.22^a^	0.0078
*Fgfr3*	1.00±0.13^a^	0.98±0.43^a^	2.26±0.85^b^	1.53±0.18^a^	0.0029
*Fgfr4*	1.00±0.18^a^	0.94±0.38^a^	1.78±0.55^b^	1.24±0.16^a^	0.0096
*Phex*	1.00±0.11^a^	0.73±0.19^b^	0	0	<0.0001
*Fgf1*	1.00±0.29^a^	0.94±0.33^a^	1.83±0.54^b^	1.03±0.11^a^	0.0030
*Fgf2*	1.00±0.36^a^	1.01±0.29^a^	2.03±0.54^b^	1.11±0.03^a^	0.0007
*Fgf23*	1.00±0.22^a^	0.53±0.21^b^	23.12±5.48^c^	16.72±0.67^d^	<0.0001
*Dmp1*	1.00±0.26^a^	0.56±0.15^b^	1.53±0.36^c^	0.84±0.14^a,b^	0.0001
*Mepe*	1.00±0.14^a^	0.42±0.11^b^	1.65±0.23^c^	0.32±0.10^b^	<0.0001
*Osteopontin*	1.00±0.19^a^	1.25±0.11^a^	2.14±0.34^b^	0.95±0.32^a^	<0.0001
*Osteocalcin*	1.00±0.12^a^	1.01±0.13^a^	1.41±0.14^b^	0.79±0.22^a^	<0.0001
*Galnt3*	1.00±0.35^a^	1.01±0.38^a^	2.00±0.91^b^	1.02±0.20^a^	0.0217
*Mmp13*	1.00±0.13^a^	1.31±0.47^a^	2.57±0.37^b^	1.41±0.46^a^	<0.0001
*Wnt10b*	1.00±0.19^a^	0.64±0.18^a^	2.69±0.85^b^	1.09±0.16^a^	0.0002
*Axin-2*	1.00±0.18^a^	0.77±0.13^a^	2.11±0.39^b^	1.08±0.12^a^	<0.0001
*Fzd2*	1.00±0.23^a^	0.97±0.31^a^	2.43±0.76^b^	1.22±0.15^a^	0.0002
*Sost*	1.00±0.17^a^	0.41±0.09^b^	0.37±0.08^b^	0.36±0.07^b^	<0.0001
*Dkk1*	1.00±0.44^a^	0.42±0.10^b^	0.61±0.12^b^	0.42±0.06^b^	0.0047
*OPG*	1.00±0.21^a^	0.77±0.11^a^	1.36±0.22^b^	0.79±0.14^a^	0.0002
*RanKL*	1.00±0.28^a^	0.84±0.19^a^	1.36±0.28^b^	0.90±0.15^a^	0.0117
**Osteoclast**					
*Trap*	1.00±0.27^a^	0.82±0.21^a^	0.96±0.32^a^	0.84±0.14^a^	0.5591
*Mmp*9	1.00±0.33^a^	0.87±0.21^a^	1.35±0.13^b^	0.89±0.14^a^	0.0072
**Chondrocyte**					
*Collagen II*	1.00±0.29^a^	0.74±0.21^a^	3.66±1.92^b^	1.24±0.52^a^	0.0015
*VegfA*	1.00±0.35^a^	1.07±0.36^a^	1.26±0.53^a^	0.95±0.21^a^	0.6095
**Adipocyte**					
*PPARγ*	1.00±0.23^a^	0.91±0.16^a^	1.22±0.27^a^	1.02±0.15^a^	0.1037
*aP2*	1.00±0.17^a^	1.18±0.16^a^	1.19±0.23^a^	0.98±0.56^a^	0.6206

Data are mean ±S.D. from 5–6 tibias of 6-week-old individual mice and expressed as the fold changes relative to the housekeeping gene *β-actin* subsequently normalized to control mice. Values sharing the same superscript between two groups are not significantly different at *P*<0.05.

**Table 2:** Biochemistry analysis in 6-week-old mice.

Parameters	*Fgfr1* ^flox/+^	*Fgfr1* ^Dmp1-cKO^	*Hyp*	*Hyp;Fgfr1* ^Dmp1-cKO^	*p*-value
FGF23 (Intact, pg/ml)	155±70^a^	67±23^a^	3607±870^b^	1099±272^c^	<0.0001
FGF23 (C-term, pg/ml)	415±94^a^	242±51^a^	6524±904^b^	1952±608^c^	<0.0001
Ratio (Intact/C-term)	0.31±0.08^a^	0.33±0.10^a^	0.65±0.11^b^	0.64±0.17^b^	<0.0001
P (mg/dl)	9.6±1.34^a^	9.8±0.63^a^	6.0±1.29^b^	9.3±1.17^a^	<0.0001
Ca (mg/dl)	7.0±0.45^a^	6.7±0.12^a^	6.7±0.63^a^	6.9±0.33^a^	0.6382
PTH (pg/ml)	229±139^a^	170±62^a^	477±216^b^	146±72^a^	0.0078
1,25(OH)_2_D (pg/ml)	178±48^a^	163±46^a^	121±23^b^	192±34^a^	0.0083

Data are mean ±S.D. from 5–6 serum samples of 6-week-old individual mice. Values sharing the same superscript between two groups are not significantly different at *P*<0.05.

We also observed significant reductions in *Sost* and dickkopf WNT signaling pathway inhibitor 1 *(Dkk1)* but not frizzled class receptor 2 (*Fzd2*), which are antagonist of the Wnt signaling; however we failed to see an increase in either *Wnt10b* or *Axin-2* in the *Fgfr1* ^Dmp1-cKO^-null mice. Loss of *Fgfr1* in osteocytes had no effect on osteoclast, chondrocyte, or adipocyte transcripts in bone (Table 1). There were no differences in the Fgf2 protein expression between control and *Fgfr1* ^Dmp1-cKO^-null mice (Figure 2B and 2C).

In spite of this reduction in FGF23, there were no significant changes in serum PTH, 1,25(OH)_2_D, phosphorus, and calcium levels in *Fgfr1* ^Dmp1-cKO^-null mice compared with age-matched control mice (Table 2). As expected due to the inclusion of an *Fgfr1* null allele in the conditional knockout strategy, *Fgfr1* transcripts were reduced by ∼50% reduction in kidney of *Fgfr1* ^Dmp1-cKO^-null mice. However, there were no significant differences in the FGF23-regulated genes, including *Npt2a*, *Npt2c*, *Cyp24a1* and *Cyp27b1*, *or* α-*Klotho* transcripts in the kidney of *Fgfr1* ^Dmp1-cKO^-null mice (Table 3).

**Table 3:** Gene-expression profiles in kidney in 6-week-old mice.

Gene	*Fgfr1* ^flox/+^	*Fgfr1* ^Dmp1-cKO^	*Hyp*	*Hyp;Fgfr1* ^Dmp1-cKO^	*p*-value
*Fgfr1*	1.00±0.11^a^	0.50±0.09^b^	1.04±0.08^a^	0.50±0.06^b^	<0.0001
*Npt2a*	1.00±0.14^a^	1.04±0.30^a^	0.48±0.15^b^	0.83±0.21^a^	0.0013
*Npt2c*	1.00±0.08^a^	0.99±0.09^a^	0.76±0.28^b^	0.98±0.32^a^	0.2417
*Klotho*	1.00±0.41^a^	1.41±0.29^a^	0.46±0.17^b^	0.92±0.30^a^	0.0008
*Cyp24a1*	1.00±0.22^a^	0.60±0.34^a^	1.57±0.54^b^	0.84±0.27^a^	0.0049
*Cyp27b1*	1.00±0.17^a^	1.25±0.22^a^	0.67±0.21^b^	0.96±0.15^a^	0.0014

Data are mean ±S.D. from 5–6 kidneys of 6-week-old individual mice and expressed as the fold changes relative to the housekeeping gene *β-actin* subsequently normalized to control mice. Values sharing the same superscript between two groups are not significantly different at *P*<0.05.

### Phex-mutant Hyp mice exhibit elevated FGF23 expression, hypophosphatemia and rickets/osteomalacia

*Hyp* mice with inactivating *Phex* mutations have very high production of FGF23 in osteocytes and markedly elevated circulating FGF23 levels leading to hypophosphatemia and abnormalities in vitamin D metabolism, as well as impaired mineralization of cartilage and bone leading to rickets and osteomalacia [31], [39], [47]. These features were present in *Hyp* mice in this study. *Hyp* mice derived from the current breeding strategy were considerably smaller, had significantly lower body weight, and demonstrated reductions in tail and femur lengths due to the presence of rickets/osteomalacia (Figure 2A and 3A). Micro-CT 3D image analyses of the femur of *Hyp* mice revealed that loss of Phex function resulted in shortened bone length, widened growth plate and reduced cortical or trabecular bone structural parameters, consistent with the presence of rickets and osteomalacia. Consistent with the abnormal mineralization in Hyp mice, histological evaluation revealed a reduction in trabecular bone volume, cortical thickness, and mineral apposition rate as well as an apparent increase of un-mineralized osteoid in both trabecular and cortical bone areas (Figure 3 and 4).

Bone samples from *Hyp* mice showed a 23-fold increase of *Fgf23* transcripts (Table 1) and western blot analysis showed a 7-fold increment in FGF23 protein levels in bone (Figure 2B and 2C). *Hyp* bone also showed a significant increase in other osteocyte-specific markers, including *Dmp1* and *Mepe*. Consistent with the observations in *Hyp* osteocytic cells [55], *Fgf1, Fgf2, Fgfr2, Fgfr3, and Fgfr4* as well as *Osteopontin*, *Osteocalcin*, *Galnt3*, *Mmp13*, *OPG*, *and RankL* mRNA levels were also increased in bone derived from *Hyp* mice (Table 1). In addition, *Hyp* mouse bone exhibited an increase in *Fzd2*, *Wnt10b*, and *Axin-2* expression, as well as suppression of *Sost* and *Dkk1*, and *Collagen II* and *Mmp9* were also increased in *Hyp* bone. We also observed more than 2-fold increase of Fgf2 protein expression in *Hyp* mice compared to control *Fgfr1* ^flox/+^ mice (Figure 2B and 2C).

Similar to previous reports [39], [47], *Hyp* mice displayed the anticipated elevations in serum Fgf23 concentrations (∼18-fold) that were associated with significant reductions in serum phosphate. There was also evidence for the previously reported diminished degradation of FGF23 in *Hyp* mice, as evidenced by alterations in the ratio of intact FGF23 to total FGF23, which was 0.31 in control mice and 0.65 in *Hyp* mice (Table 2). In addition, serum PTH levels were significantly elevated in *Hyp* mice, whereas 1,25(OH)_2_D levels were significantly decreased in *Hyp* mice compared with age-matched control mice. Consistent with known effects of FGF23 on the kidney, *Hyp* mice exhibited reduction of *Npt2a* and *Klotho* transcripts (Table 3), increments in *Cyp24a1* and decrements in *Cyp27b1* (Table 3).

### Osteocyte specific deletion of *Fgfr1* in Phex-mutant Hyp mice partially rescues the hypophosphatemic rickets phenotype

The conditional deletion of *Fgfr1* in osteocytes of compound *Hyp*;*Fgfr1* ^Dmp1-cKO^-null mice resulted in partial rescue of the *Hyp* gross phenotype. In this regard, the tail length, a marker of rickets, was 2″ shorter in *Hyp* mice compared to controls, but was only 0.5″ shorter in compound *Hyp*;*Fgfr1* ^Dmp1-cKO^-null mice compared to *Fgfr1^flox/+^* control mice. Similarly, compound *Hyp*;*Fgfr1* ^Dmp1-cKO^-null mice displayed body weight and femur lengths intermediate to those of *Hyp* and control mice (Figure 2A and 3A).

More importantly, both the elevated serum FGF23 and hypophosphatemia in *Hyp* mice were significantly improved in compound *Hyp*;*Fgfr1* ^Dmp1-cKO^-null mice. Conditional deletion of *Fgfr1* in *Hyp* osteocytes resulted in about 70% reduction in serum FGF23 (from 3607 to 1099 pg/ml) and about 50% increase in serum phosphorus (from 6.0 to 9.3 mg/dl, values not significantly different from age-matched control mice). Ablation of *Fgfr1* in *Hyp* osteocytes also resulted in reductions of PTH to the normal range, changes associated with significant increments of serum 1,25(OH)_2_D and phosphate concentrations. We observed no significant differences in serum calcium concentrations across the four genotypes (Table 2). Serum FGF23 and phosphorus concentrations (207±84 pg/ml and 9.8±0.63 mg/dl, respectively) were not significantly different in *Fgfr1* ^flox/null^ (equivalent to *Fgfr1* ^+/null^) mice compared to *Fgfr1* ^flox/+^ controls (155±70 pg/ml and 9.6±1.34 mg/dl, respectively) (Table 3). In *Hyp*;*Fgfr1* ^flox/null^ (equivalent to *Hyp*;*Fgfr1* ^+/null^) mice, however, we observed an intermediate reduction of serum Fgf23 (1881±230 pg/ml) and a corresponding increment of serum phosphorus (7.2±0.84 mg/dl) compared to *Hyp*;*Fgfr1* ^Dmp1-cKO^-null mice, indicating a gene dosage effect of Fgfr1 reductions in osteocytes on the *Hyp* phenotype.

In contrast to the nearly 70% reduction in both bone FGF23 proteins and circulating FGF23 concentrations in *Hyp*;*Fgfr1* ^Dmp1-cKO^-null mice, conditional deletion of *Fgfr1* in *Hyp* osteocytes resulted in only a 28% reduction of *Fgf23* transcripts in bone of *Hyp*;*Fgfr1* ^Dmp1-cKO^-null compared with *Hyp* mice (Figure 3C &3D and Table 1 & 2). In *Hyp* mice the ratio of intact and C-terminal FGF23 in serum was consistent with impaired FGF23 degradation in *Hyp* mice, as previously described [56], [57], however conditional deletion of *Fgfr1* in *Hyp* osteocytes did not affect the ratio of intact and C-terminal Fgf23 in the compound *Hyp*;*Fgfr1* ^Dmp1-cKO^-null mice (Table 2).

Conditional deletion of *Fgfr1* in *Hyp* osteocytes normalized *Npt2a* and *Klotho* expression in the kidney. The alterations in *Cyp27b1* and *Cyp24* transcripts that regulate vitamin D metabolism were also corrected in the kidney from compound *Hyp*;*Fgfr1* ^Dmp1-cKO^-null mice. These results are consistent with an effect of reduced circulating FGF23 to increase serum phosphate and 1,25(OH)_2_D levels in the compound *Hyp*;*Fgfr1* ^Dmp1-cKO^-null mice.

In addition to the improvement in serum biochemistries in *Hyp* mice by conditional deletion of *Fgfr1* in osteocytes, there was a partial rescue of the hypophosphatemic rickets phenotype in the compound *Hyp*;*Fgfr1* ^Dmp1-cKO^-null mice. Although trabecular bone volume was not corrected in compound mutant mice skeletal abnormalities remained, consistent with an intrinsic mineralization defect caused by *Phex* mutations, as previously described [58]; there was a partial correction of the length of femur and cortical thickness in the compound *Hyp*;*Fgfr1* ^Dmp1-cKO^-null mice. Growth plate abnormalities were also qualitative improved, as evidenced by a more organized structure. In addition, the area of un-mineralized osteoid in cortical bone was reduced and an increase in mineral apposition rate was observed in *Hyp*;*Fgfr1* ^Dmp1-cKO^-null mice compared to age-matched *Hyp* mice (Figure 3 and 4).

*Fgf1*, *Fgf2*, *Fgfr2*, *Fgfr3*, *Fgfr4* as well as *Dmp1*, *Mepe*, *Osteopontin*, *Osteocalcin*, *Galnt3*, *Mmp13*, *Mmp9*, and Collagen II message levels were elevated in *Hyp* mice; and all of these transcripts were reduced in compound *Hyp*;*Fgfr1* ^Dmp1-cKO^-null mice, consistent with partial healing of bone and cartilage (Table 1). Conditional deletion of *Fgfr1* in *Hyp* osteocytes also partially corrected the observed abnormalities of the Wnt signaling pathway, including reductions in *Wnt10b*, *Axin-2*, and *Fzd2*. However, *Sost* and *Dkk1* remained suppressed in *Hyp*;*Fgfr1* ^Dmp1-cKO^-null mice.

### *Fgfr1* regulates FGF23 gene transcription *in vitro* via a MAPK dependent pathway

**Figure 5:**
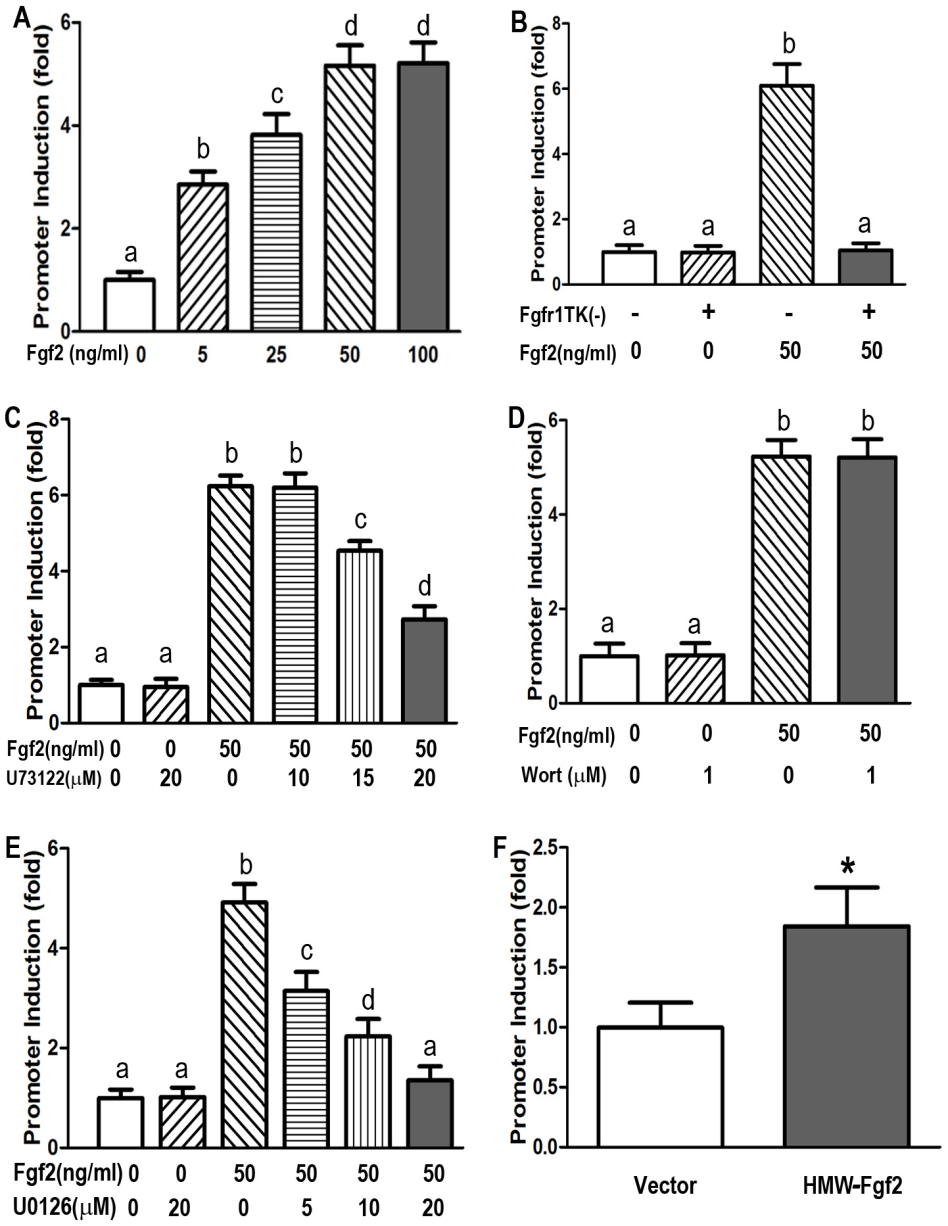
Enhancement of mouse *FGF23* promoter activity by FGF2 via FGFR1 signaling pathway. MC3T3-E1 cells were co-transfected with various plasmids and treated with different drugs as described in Material and Methods. (A) Dose-dependent stimulation of mouse *FGF23* promoter activity by recombinant FGF2 (5∼100 ng/ml); (B) Dominate-negative *FGFR1(TK−)* constructs blocked the stimulatory effect of recombinant FGF2 (50 ng/ml) on mouse *FGF23* promoter activity; (C) Effect of PLCγ inhibitor (U73122) on FGF2-induced *FGF23* promoter activity; (D) Effect of PI3K inhibitor Wortmannin (Wort) on FGF2-induced *FGF23* promoter activity; (E) Dose-dependent inhibition of FGF2-induced *FGF23* promoter activation by MAPK inhibitors (U0126). (F) Overexpression of HMW-*Fgf2* constructs stimulated mouse *FGF23* promoter activity. Data are expressed as the mean ± S.D. from triple three independent experiments. Values sharing the same superscript in different groups are not significantly different at *P*<0.05. * indicates significant difference from control vector group.

To test the role of FGFR1 activation on FGF23 gene transcription, we co-transfected MC3T3-E1 osteoblasts with a mouse *Fgf23* 0.6 kb promoter-lucifierase reporter construct (p0.6 kb-*Fgf23*-luciferase) and either a wild-type *FGFR1* or a dominant negative *FGFR1(TK-)* cDNA construct [59], [60]. Addition of FGF2 (5∼100 ng/ml), dose dependently, stimulated *Fgf23* promoter activity in MC3T3-E1 osteoblasts co-transfected with *FGFR1* (Figure 5A), achieving a maximal response at an FGF2 of 50 ng/ml. Co-transfection of the dominant negative *FGFR1(TK−)* completely inhibited FGF2 stimulation of FGF23 promoter activity in MC3T3-E1 osteoblasts overexpressing FGFR1 (Figure 5B). To investigate which of the FGFR1-dependent signaling pathway (i.e., PI3K/AKT, RAS/MAPK, and PLCγ) mediating the actions of FGFR1 on *FGF23* gene transcription, we investigated the effects of a Wortmannin, a PI3K inhibitor, U73122, a PLCγ inhibitor, and U0126, an inhibitor of MAPK pathway, on FGF2-stimulated FGF23 promoter activity. Neither Wortmannin (1 µM, Figure 5C) or low dose of the PLCγ inhibitor U73122 (10 µM, Figure 5D) inhibited the effects of FGF2 to stimulate FGFR1-dependent activation of FGF23-promoter activity; however, high dose of PLCγ inhibitor U73122 (15∼20 µM, Figure 5D) and U0126, which inhibits both MEK1 and MEK2, exhibited a dose-dependent inhibition of FGFR1-mediated stimulation of FGF23 promoter activity (Figure 5E). In addition, we observed that overexpression of HMW-*FGF2*, which activates intracrine dependent FGFR1 signaling [61], stimulated FGF23 promoter activity (Figure 5F).

### *Fgfr1* signaling regulates FGF23 protein expression in vitro via a PI3K-AKT dependent pathway

**Figure 6:**
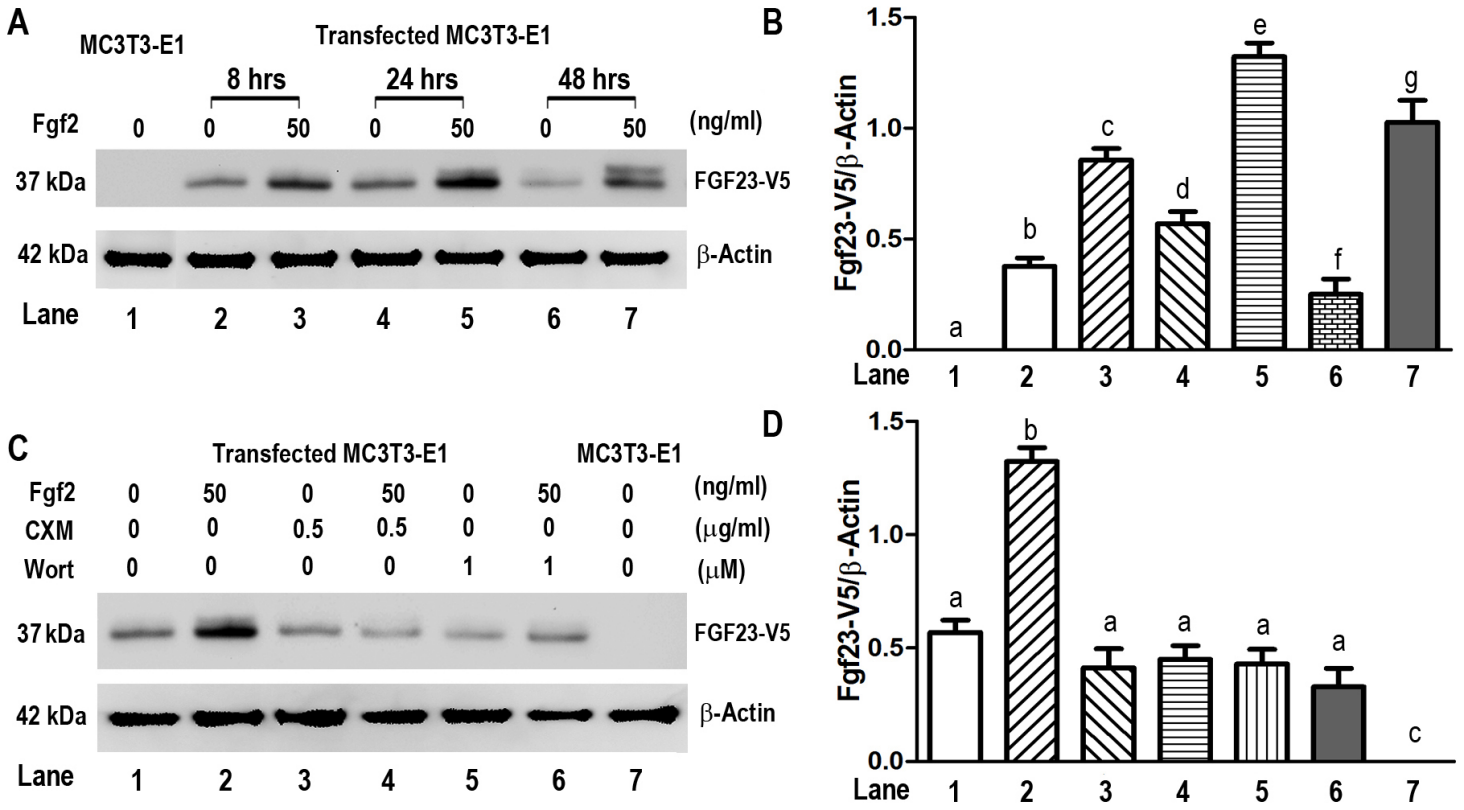
Translational control of human FGF23-V5 protein expression by FGF2 via FGFR1 signaling pathway. MC3T3-E1 cells were co-transfected with pcDNA3.1-*FGFR1* expression plasmids and human pcDNA3.1-FGF23-V5-His expression plasmids and treated with different drugs as described in Material and Methods. (A and B) Time-dependent stimulation of human FGF23-V5 protein expression by recombinant FGF2 (50 ng/ml); (C and D) Effect of PI3K inhibitor Wortmannin (Wort) and protein synthesis inhibitor Cycloheximide (CXM) on FGF2-induced human FGF23-V5 protein expression. Both Wortmannin and Cycloheximide completely blocked FGF2-induced FGF23 protein increase. Data are expressed as the mean ± S.D. from triple three independent experiments. Values sharing the same superscript in different groups are not significantly different at *P*<0.05.

Since we found disproportionate reductions in serum circulating FGF23 protein and bone *Fgf23* mRNA levels in compound *Hyp*;*Fgfr1* ^Dmp1-cKO^ mice, and FGFR1 is known to enhance recruitment of RNA to polysomes to increase in protein expression in other systems [62], we examined if Fgfr1 regulate *Fgf23* protein expression through an effect to stimulate mRNA translation. For these studies we transfected human *FGF23*-*V5-His* cDNA expression plasmid into mouse MC3T3-E1 osteoblasts and assessed epitope tagged FGF23 protein production before and after stimulation with FGF2. We found that FGF2 resulted in a time-dependent induction of *FGF23*-V5-His protein expression, achieving a maximal 3-fold stimulation at 24 hours after Fgf2 stimulation (Figure 6A and 6B). Both the PI3K inhibitor Wortmannin and protein synthesis inhibitor Cycloheximide completely blocked FGF2-induced FGF23 protein increase (Figure 6C and 6D), consistent with an effect of FGF2 to stimulate FGF23 protein translation through activation of PI3K/Akt pathway.

## Discussion

Osteocytes, the most abundant cells in bone, play a central role in regulating bone remodeling and mineralization through the release of paracrine factors and systemic phosphate and vitamin D metabolism through the release of the hormone FGF23 [63]. FGFR1 is known to be involved in the regulation of bone development and remodeling [64], [65]. Inactivating mutations of *FGFR1* in autosomal dominant Kallmann syndrome [66] and the Pfeiffer craniosynostosis [67] and conditional deletion of *Fgfr1* gene in osteo-chondro-progentitors using *Col1a1(2.3)*-Cre enhances osteoblast differentiation and endochondral bone formaton [65], suggesting that FGFR1 has osteoblast-stage specific effects. The specific role of FGFR1 in osteocytes, however, has not been previously defined. The current study is the first to selectively ablate *Fgfr1* gene in osteocytes in bone.

We found that *Dmp1-*Cre-mediated loss of *Fgfr1* in osteocytes resulted in selective alterations of multiple genes involved in bone remodeling and mineral metabolism. *Fgfr1* ^Dmp1-cKO^-null mice exhibited reductions in *Fgf23* transcripts in bone and circulating levels of FGF23. In addition, targeted disruption of *Fgfr1* in osteocytes resulted in reductions of *Dmp1* and *Mepe*, two members of the SIBLING family of proteins that are expressed in osteocytes as well as *Phex*, an endopeptidase, that are involved in the regulation of bone mineralization. We also found in the *Fgfr1* ^Dmp1-cKO^-null mice evidence for reductions in *Sost* and *Dkk1*, which are produced by osteocytes and function as antagonists of the Wnt-signaling pathway, which is an important anabolic signal for bone. In contrast, *Fgfr1* ^Dmp1-cKO^-null mice exhibited no changes in more general markers of osteoblast differentiation, such as *Osteopontin and Osteocalcin, and* bone expression of *Wnt10b* and *Axin-2* were decreased, not increased, in conditional the *Fgfr1* ^Dmp1-cKO^-null mice, as would be expected from reductions in *Sost and Dkk1*. In spite of these changes in osteocyte gene expression, we observed no abnormalities of bone structure or mineralization (*i.e.*, no changes in bone volume or mineral apposition rates) in the *Fgfr1* ^Dmp1-cKO^-null mice under normal conditions. This may be due to the complexity of FGFR1 signaling in bone and cross-talk between downstream molecular targets affecting bone remodeling [68] that may have offset the effects of the loss-of-FGFR1 in osteocytes.

Indeed, additional pathological perturbations caused by *Phex* mutations in *Hyp* mice uncovered a more evident role of *Fgfr1* in osteocytes on bone structure and gene expression [29]. We found that conditional deletion of *Fgfr1* in osteocytes of *Hyp* mice decreased *Fgf23* transcripts in bone, as well as a lead to a striking reduction in circulating FGF23 levels in compound *Hyp*;*Fgfr1* ^Dmp1-cKO^-null mice. The reductions in FGF23 in *Hyp* mice partially rescued the hypophosphatemic rickets phenotype, as evidenced by increased serum phosphate levels, improved bone parameters and bone-related gene expression as well as improved kidney gene expression and 1,25(OH)_2_D metabolism. In addition, alterations in *Fgf1* and *Fgf2*, as well as *Fgfr2*, *Fgfr3*, and *Fgfr4* expression that are known to be increased in osteocytes derived from *Hyp* bone [55] were normalized in *Hyp*;*Fgfr1* ^Dmp1-cKO^-null mice (Table 1), indicating that Fgfr1 regulates other members of the FGF/FGFR signaling family in *Hyp* osteocytes. Since FGF23 has dose-dependent effects on the kidney [69], [70], the 3-fold reduction in circulating FGF23 in *Hyp*;*Fgfr1* ^Dmp1-cKO^-null mice likely accounts for the improvement in the hypophosphatemic rickets phenotype, whereas the persistent elevations of FGF23 would explain the residual hypophosphatemia and bone abnormalities in *Hyp*;*Fgfr1* ^Dmp1-cKO^-null mice. Alteration in matrix mineralization caused by loss of *Phex* function are persistent [41], [58], [71] and could contribute to persistent skeletal abnormalities are present in the compound *Hyp*;*Fgfr1* ^Dmp1-cKO^null mice.

Studies of FGF23 gene transcription and translation in osteoblast cultures confirmed an important role of FGFR1 in regulating FGF23 production. In this regard, we demonstrated that a dominant negative *FGFR1(TK−)* construct inhibits FGF2-mediated activation of FGF23 gene transcription in cultured osteoblasts. Interestingly, inhibition of MAPK and PLCγ pathways, but not PI3K/Akt pathways, blocked FGFR1 stimulation of FGF23-promoter activity in cultured osteoblasts. MAPK regulation of FGF23 transcription is consistent with the recent findings that activating somatic mutations of RAS causes FGF23-mediated hypophosphatemia in humans [72] and that ERK1/2 activation is involved in FGFR-mediated FGF23 transcription in UMR-106 osteoblasts *in vitro* [40]. Transfection of a HMW-FGF2 cDNA, an intracrine activator of FGFR1, also stimulated FGF23 promoter activity in osteoblast cultures. Thus, both autocrine/paracrine and intracrine activation of FGFR1 regulates FGF23 expression in osteoblasts.

Our studies also identified a possible role of FGFR1 in the post-transcriptional regulation of FGF23. In this regard, reductions in circulating Fgf23 levels were greater than the decrease in *Fgf23* mRNA expression in bone of *Hyp*;*Fgfr1* ^Dmp1-cKO^-null mice, indicating a possible role of FGFR1 in regulation of *Fgf23* mRNA translation. Indeed, we found that FGF2-FGFR1 signaling regulates FGF23 protein translation *in vitro* via a PI3K-AKT dependent pathway. These results are consistent with prior reports showing translational control role FGFR1 signaling in cancer and smooth muscle cells [62], [73], [74]. The dual transcriptional and post-transcriptional control of FGF23 by FGFR1 may explain discrepancies between FGF23 message and circulating levels of this hormone [75], as well as provide another therapeutic target, along with regulation of transcription and degradation [76], to modify circulating FGF23 levels in disease states.

Previous data have implicated FGFR1 signaling in the regulation of FGF23 expression, but these observations lack the cell- and organ- specificity of our studies [77], [78]. For example, pharmacological inhibition of FGFR1 *in vivo* in *Hyp* mice [78] results in inhibition of both FGF23 end-organ effects as well as variable effects on FGF23 production by bone. Similarly, Y372C missense gain-of-function mutation in *FGFR1* in osteoglophonic dysplasia (OGD) and systemic administration of activating antibodies to FGFR1 in mice elevate circulating FGF23 levels [79], but have the confounding effects of activation of FGFRs in both bone and kidney. Thus, use of non-specific inhibitors of FGFR1 or FGFR1 activating antibodies *in vivo* are limited by generalized effects that prevent distinguishing between actions on FGFR1 in osteocytes or indirect effects through other actions on FGFRs in the kidney or other tissues. In contrast, the selective deletion of *Fgfr1* in osteocytes provides *in vivo* confirmation of prior *in vitro* studies showing that pan-FGFR inhibitors directly suppress FGF23 expression in *Hyp*-derived osteoblasts and FGF1 and FGF2-mediated FGFR1 activation directly stimulates FGF23 promoter activity in osteoblasts cultures [39], [77].

Perhaps more importantly, we have discovered that the evolutionarily linked canonical (or autocrine/parcrine) and intracellular (or intracrine) FGFs are also physiologically coupled to the hormone-like FGF23 in bone through FGFR1-dependent mechanisms. FGF1 and FGF2 (both low and HMW forms), produced by the osteoblast lineage and stored in bone matrix, are increased in *Hyp* bone [37]–[39]. Osteocytes also participate in a peri-cellular demineralization process called osteolytic osteolysis that involves matrix degradation and growth factor activation by enzymes, such as Matrix metalloproteinase-9 and 13 (MMP-9 and –13), which are also increased in *Hyp* mice [80]. It is tempting to speculate that either the local release of stored FGFs from the extracellular matrix, or other factors regulating, FGF1, FGF2 and HMW-FGF2, are regulating osteocyte functions through FGFR signaling. Further studies are needed to define how alterations in bone metabolism and mineralization caused by *Phex* mutations regulate FGF ligands and FGFR signaling pathways in osteocytes. Since hormonal-like FGFs emerged early in vertebrate evolution, our findings raise the possibility that the mineralized skeleton developed a means to coordinate local and systemic functions by the linkage of autocrine/paracrine and intracrine FGF signaling with hormonal FGF23.

Our study has several limitations. First, conditional deletion of *Fgfr1* failed to completely restore FGF23 expression to normal in the compound *Hyp*;*Fgfr1* ^Dmp1-cKO^-null mice. The persistent elevations of FGF23 might be due to incomplete ablation of *Fgfr1* in osteocytes or continued production of FGF23 from osteoblasts not affected by *Dmp1*-Cre (*e.g.*, 30% of *Fgfr1* transcripts persisted in bone of *Fgfr1* ^Dmp1-cko^-null mice and *Dmp1*-Cre targets ∼1% of osteoblasts in long bone). Alternatively, FGFR1-independent pathways known to regulate FGF23 expression may account for the elevations of FGF23. The possibility that other FGFRs in osteocytes might mediate the residual stimulation of Fgf23 in *Hyp* mice appears unlikely, since we observed a decrease in *Fgfr2*, *Fgfr3*, and *Fgfr4* in *Hyp*;*Fgfr1* ^Dmp1-cKO^-null mice. In addition, prior studies indicate that conditional deletion of *Fgfr2* in mice leads to no abnormalities of serum FGF23 or phosphate [81], and global loss of *Fgfr3* and *Fgfr4* enhances, rather than inhibits, FGF23 expression in bone, due to impaired end organ sensing of FGF23 by the kidney [82], [83]. Second, although *Dmp1*-Cre had no kidney expression, to achieve the 70% reduction of *Fgfr1* expression in osteocytes using *Dmp1*-Cre required using a strategy that paired a flox with a null *Fgfr1* allele, which led to a 50% reduction in *Fgfr1* expression in the kidney, and other tissues. Meaningful conclusions can be made about FGFR1 function in bone, however, because heterozygous *Fgfr1* ^flox/null^ (equivalent to *Fgfr1* ^+/null^) mice had no demonstrable renal phenotype and the reduction in FGF23 expression was dose-dependently related to reductions in *Fgfr1* expression in bone. We lack evidence that end-organ resistance to FGF23 actions on the kidney in *Hyp*;*Fgfr1* ^Dmp1-cKO^-null mice, since this would lead to hyperphosphatemia and elevated FGF23 levels, a phenotype different from the one observed in these mice.

In conclusion, conditional deletion of *Fgfr1* in osteocytes from *Hyp* mice reduced circulating FGF23 concentrations through regulation of both the transcription and translation of FGF23. These studies establish a function of FGFR1 in osteocytes and show a physiological coupling between canonical FGFRs and hormone-like FGFs that may provide a mechanism to link local regulation of bone metabolism with systemic phosphate and vitamin D homeostasis. This evolutionary relation may permit local changes in bone metabolism regulated by FGFs-FGFRs to communicate with the kidney to permit FGFR1 regulation of bone formation and mineralization to be coordinated with phosphate absorption and/or vitamin D metabolism to match the bone needs for mineral with the renal handling of these minerals.
